# Spatial variation of cardiovascular mortality in Cali, Colombia, between 2010 and 2017

**DOI:** 10.1186/s12889-022-14907-x

**Published:** 2023-03-31

**Authors:** Luisa M. Murillo, Carolina Ramírez, Mercedes Andrade-Bejarano, Guillermo Perlaza, Lena Barrera

**Affiliations:** 1 School of Statistics, Universidad del Valle, Calle 13 # 100-00, Edificio E56, Ciudad Universitaria Meléndez, Cali, Colombia; 2 Division of Health, Municipality of Cali, Cali, Colombia; 3 School of Medicine and School of Public Health, Universidad del Valle, Calle 4B # 36‑00 Edificio 118‑ Piso 2, Oficina 214, Cali, Colombia; 4 Department of Primary Care and Public Health, School of Public Health, Imperial College London, London, UK

**Keywords:** Cardiovascular mortality, Spatial aggregation, Mortality rate

## Abstract

**Background:**

Cardiovascular disease (CVD) is the leading cause of mortality worldwide and in Colombia. The analysis of CVD mortality has been mainly relied on individual factors and rates, but occurrence is also related to contextual conditions. Understanding the distribution of CVD in a region will contribute to implement more focused-preventive and care interventions.

**Methods:**

Using the national mortality register established by the Department of National Statistics, standardized rates and spatial distribution of CVD mortality were estimated for Cali, Colombia, between 2010–2017. Global and local spatial aggregation was assessed using the Geary’s C test and for each district standardized mortality ratios smoothed by the Bayesian empirical were estimated.

**Results:**

Over the period, CVD was the main cause of mortality with 28,804 deaths accounting for 23,8% of total deaths. The global CVD mortality rate varied from 235.9 to 257.4 per 100.000 habitants, with an average increase of 9.1% in the percentage change. The main cause of mortality were hypertensive diseases following by ischemic heart diseases and stroke. The standardized mortality ratios smoothed by the Bayesian empirical method showed that the districts 7, 13, 14, 15 and 16 located at the eastern area with the lowest socio-economic strata and the district 22 at the south of the city with the highest socio-economic strata had the high risks of CVD mortality. All these areas were at the boundary of the city. The the lowest risk was observed in districts 1 and 2 at the northwest area with the upper socio-economic strata. Over the study period, a spatial autocorrelation was found in the districts 1,9 10, 11, 12, 13, 14, 15, 19, and 21 by using the Geary’s C test. The highest significant spatial association was found in the districts 1 and 21.

**Conclusion:**

Of 22 districts in Cali, the highest risk of CVD mortality was found in three at the lowest and one in the upper socio-economic strata between 2013 and 2017. Over the period a global spatial aggregation was identified due mainly to districts peripherical located suggesting that there could be contextual conditions influencing the risk. Therefore, there is a need for considering local conditions to prevent CVD mortality.

**Supplementary Information:**

The online version contains supplementary material available at 10.1186/s12889-022-14907-x.

## Introduction

Cardiovascular diseases (CVDs) are the main cause of mortality and disability-adjusted life-years worldwide [1]. Similarly, in Colombia, a low-middle income country, CVDs are the main cause of mortality, accounting for nearly 30% of total mortality in 2019 [2]. The main cause is coronary heart disease with a rate of 80.7 per 100.000 habitants in 2017 and with an increasing of 24.7% in deaths between 2009 and 2019 [2, 3]. Moreover, analysis of cardiovascular mortality from 23 high income countries shown that the constant decline of the cardiovascular mortality rate started in 1970 has been stable or even upward in some countries since 2000 [4].

The conditions leading to CVDs are heterogeneous and relying on individual and population determinants [5]. A geographical pattern for incidence and mortality of CVD within and between countries has been reported. According to the last estimation provided by the Global Burden of Disease project, in 2019, the highest age-standardized rates of cardiovascular mortality were found in Uzbekistan, Solomon Islands and Tajikistan and the lowest in France, Peru and Japan [6]. The differences are influenced by the prevalence of cardiovascular risk factors, the evolution of health care and the interventions leading to reduce the environmental exposure including pollution, access to healthy food and inequalities [5].

Risk factors for CVDs are well established and classified as modifiable and non-modifiable. The former includes conditions such as diabetes, hypertension, smoking, physical inactivity, unhealthy diet, obesity, and dyslipidaemia, and the latter includes age, male sex, and family history of premature cardiovascular disease [7]. Increases in the prevalence has been observed in Latin-American countries in the last twenty years [8] Chaix B has pointed out that the neighbourhood, social interaction, services, and physical environment are the determinants the spatial geographical distribution and related inequalities of CVDs. Therefore, Chaix has appointed that the individual characteristics could account for only up to 60% of the inequalities in cardiovascular mortality [9].

To detect the contribution of neighbourhood conditions is crucial to prevent and control CVDs. Different spatial–temporal analysis of CVDs has shown clustering occurrence related to aging, illiteracy, urban development, pollution, social deprivation, and ethnic segregation [10, 11]. Ethnic disparities have been related to health insurance coverage, access to health care and conceptions on health [12] Pollution measuring as each 1-mg/m^3^ increase in carbon monoxide, increases the risk of acute infarction by nearly 5% for those exposed over the seven days before the event [13] and similarly, those living in neighbours with lower walkability areas have 13% more cardiovascular risk than those in areas with higher walkability areas [14].

To measure cardiovascular rates at small units needs to adjust for small population [15]. Current methods such age-standarized rate (ASR) requires knowing the age-structure population of the population study which in small areas is not always available [16]. Although the standardized mortality ratio (SMR) removing age effect and allowing for comparison between areas, the results could have a high heterogeneity [15]. Therefore, for better estimations, SMR need to be smoothed. Bayesian approaches and regression models have been used to fit the estimations of SMR [15]. These approach are more suitable to measure spatial correlations and to identify clustering at small units [15].

Since 1993, Colombia has a universal health insurance with primary care framework established in 2007 [17]. Although the framework has incorporated the concept of social determinants, the framework lacks the integration of neighbourhood as a relevant condition for population health. Particularly, little is known about the interaction between areas and cardiovascular diseases. We aimed to measure the spatial aggregation of cardiovascular diseases in Cali between 2010 and 2017. The city is the third largest city of Colombia with 22 districts which differ socio-economic characteristics and the main cause of mortality is cardiovascular diseases.

## Methods

### Study region

**Fig. 1:**
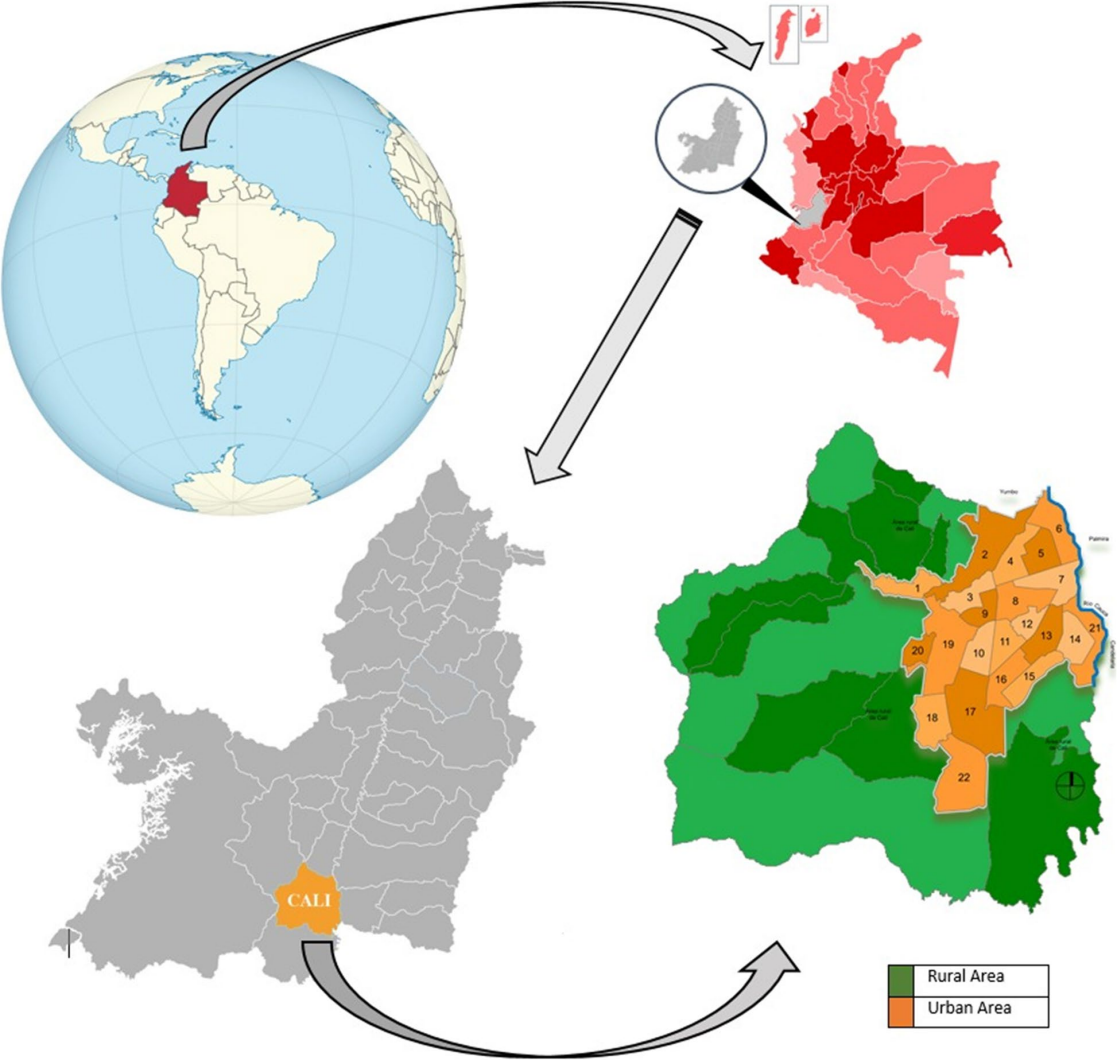
Map of Cali (Colombia) by urban area (22 districts) and rural area (15 townships)

Cali is the third largest city in Colombia, South America, with 1,822,869 inhabitants in 2018 [18]. The city is the capital of the state of Valle del Cauca located at the Colombian southwest and is divided into urban and rural areas; the urban comprises 22 districts, and the rural area 15 townships [19]. See Fig. 1.

### Data source

The data were obtained from death certificates with the basic cause of mortality registered as cardiovascular diseases with the codes I00-I99 for diseases of circulatory system based on the International Classification of Diseases version 10 (ICD-10) [20]. The certificate was filled by physicians and collected in a data base of Secretary of Health in each municipality [21]. The data was processed and transferred to the national vital statistics, and it was accessible to public [22]. For each death the following variables were analysed the district or townships of residence, age, marital status, educational level, health insurance and sex. The certificates without information on age, sex, the place of residence or those with place of residence different from Cali were excluded.

### Statistical analysis

#### Epidemiological analysis

The mortality rates of cardiovascular diseases were estimated by the direct method [16] using the 2018 population of Cali [18] as the standard population. The annual variation was estimated using the percentage of change (PC) of the rates over time [23]. The calculations were performed for each district, type of cardiovascular disease and sex.

#### Spatial analysis

The geographical unit for the spatial analysis was each district. The standardized mortality ratio of cardiovascular mortality (SMR) was calculated using the 2018 population of Cali [18]. The SMR was smoothed by means of the empirical Bayes method [24]:

$${\widetilde{\rho }}_{\left(i\right)}={\widetilde{m}}_{\left(i\right)}+{\widetilde{C}}_{\left(i\right)}\left({\widehat{\rho }}_{(i)}-{\widetilde{m}}_{\left(i\right)}\right)$$

$$={\widetilde{C}}_{\left(i\right)}{\widehat{\rho }}_{(i)}+{(1-\widetilde{C}}_{\left(i\right)}) {\widetilde{m}}_{\left(i\right)}$$

where ${\widetilde m}_{\left(i\right)}=\;\frac{\sum_{i=1}^{n_{(i)}}{\widehat p}_{(i)}E_{(i)}}{\sum_{i=1}^{n_{(i)}}E_{(i)}}$ is the weighted sample mean, 𝑛_(i)_ is the number of nearest neighbours to district *i*.

${\widehat p}_{\left(i\right)}=\frac{z_{(i)}}{E_{(i)}}$, is the maximum likelihood estimator of ${\rho }_{i}$; ${z}_{i}$ is the number of deaths in district *i* and ${E}_{i}$ number of expected deaths in district *i*.

${\widetilde C}_{\left(i\right)}$ is the shrinkage factor; ${\widetilde{C}}_{\left(i\right)}=\frac{{\widetilde{v}}_{(i)}}{{\widetilde{v}}_{(i)}+\frac{{\widetilde{m}}_{(i)}}{{E}_{(i)}}}$, where ${\widetilde v}_{\left(i\right)}=s_{(i)}^2-\frac{{\widetilde m}_{\left(i\right)}}{{\overline E}_{(i)}}=\frac{\sum_{i=1}^{n_{\left(i\right)}}E_i\left({\widehat\rho}_{\left(i\right)}-{\widetilde m}_{\left(i\right)}\right)^2}{\sum_{i=1}^{n_{\left(i\right)}}E_{(i)}}-\frac{{\widetilde m}_{\left(i\right)}}{{\overline E}_{(i)}}$, ${\overline E}_{(i)}=\frac{\sum_{i=1}^{n_{(i)}}\;E_{(i)}}{n_{(i)}},$  

The inverse-distance weighting ${w}_{ij}={d}_{ij}^{-\gamma }$ was calculated for the analysis of spatial autocorrelation [25]. For the spatial autocorrelation analysis global index and local indicator of spatial association (LISA) were obtained.

### Global Test

The Global Geary´s C test was estimated being this an appropriate index when there are extreme values as it was observed in the current analysis [26].

The null hypothesis of this test is:

- Ho: The rate risk is constant over the study region; therefore, individuals have the same probability of developing the disease regardless of location, i.e., no spatial autocorrelation

The Global Geary’s C test was estimated as [25]:

$$C=\frac{n-1}{2\sum_{i=1}^n({SMR}_i-\overline{SMR})^2}\frac{\sum_{i=1}^n\sum_{j=1}^nw_{ij}({SMR}_i-{SMR}_j)^2}{\sum_{i=1}^n\sum_{j=1}^nw_{ij}},$$

Where:

- ${SMR}_i,SMR_j=$ are standardized mortality ratios in district *i* and *j*, respectively.
- $\overline{SMR }=$ Overall mean of standardized mortality ratios
- ${w}_{ij}=$ inverse-distance weighting between districts *i* and *j*
- $n=$ total districts in Cali

### Local Indicator of Spatial Association (LISA)

Local indicators of spatial association (LISA) were obtained to estimate a local measure of similarity among each district and its nearest neighbours. The null hypothesis of this indicator is the absence of spatial correlation in the neighbourhood of the district *i*. The indicators used was Local Geary´s *C* test [27]:

$$C_{\left(i\right)}=\frac{\sum_{j=1}^nw_{ij}({SMR}_i-{SMR}_j)^2}{\displaystyle\frac{\sum_{i=1}^n({SMR}_i-\overline{SMR})^2}n}$$

The data was processed using R software, version 3.6.0, GeoDa, version 1.14.0.0 and Epidat, version 4.2.

## Results

From 2010–2017, 121,254 deaths registered with place of residence Cali were identified. Of them 28.804 (23.76%) were classified as cardiovascular diseases, 14.834 (51.50%) were women and 13.969 (48.50%) were men. Premature cardiovascular deaths were higher in men, but the opposite trend was observed in those aged 80 years and older. In 2010, among 12.763 deaths registered, 8.154 (63.9%) were due non-communicable diseases (NCDs) and CVDs accounted for most of them with 3.900 (39.2%). Similarly, in 2017, of 15.729 deaths, 10.161 (64.6%) were due to NCDs and 4189 (41.2%) were attributed to cardiovascular diseases. (Table S1, Supplementary).

### Changes in the mortality rates during the period 2010–2017

**Fig. 2:**
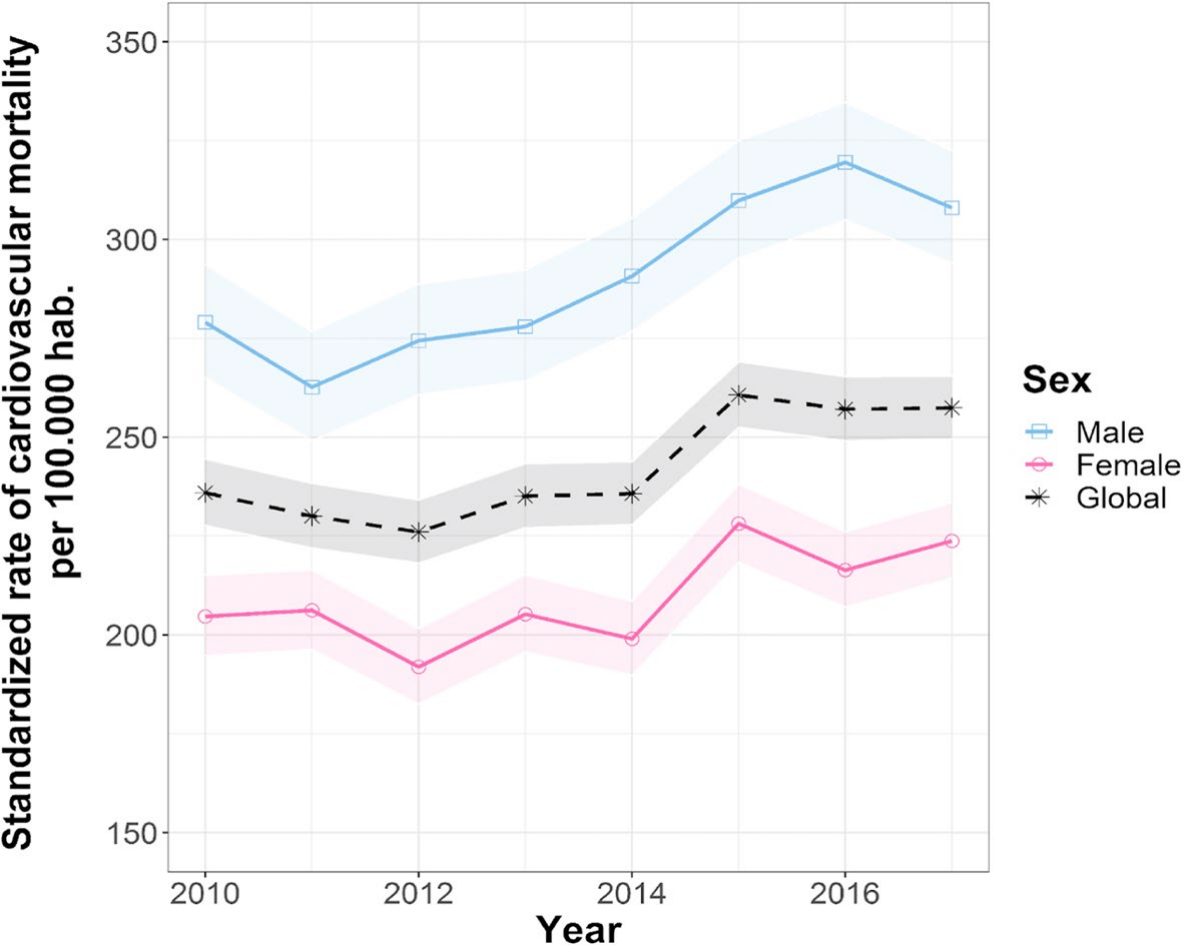
The age-standardized rate of cardiovascular in Cali between 2010 and 2017

**Fig. 3:**
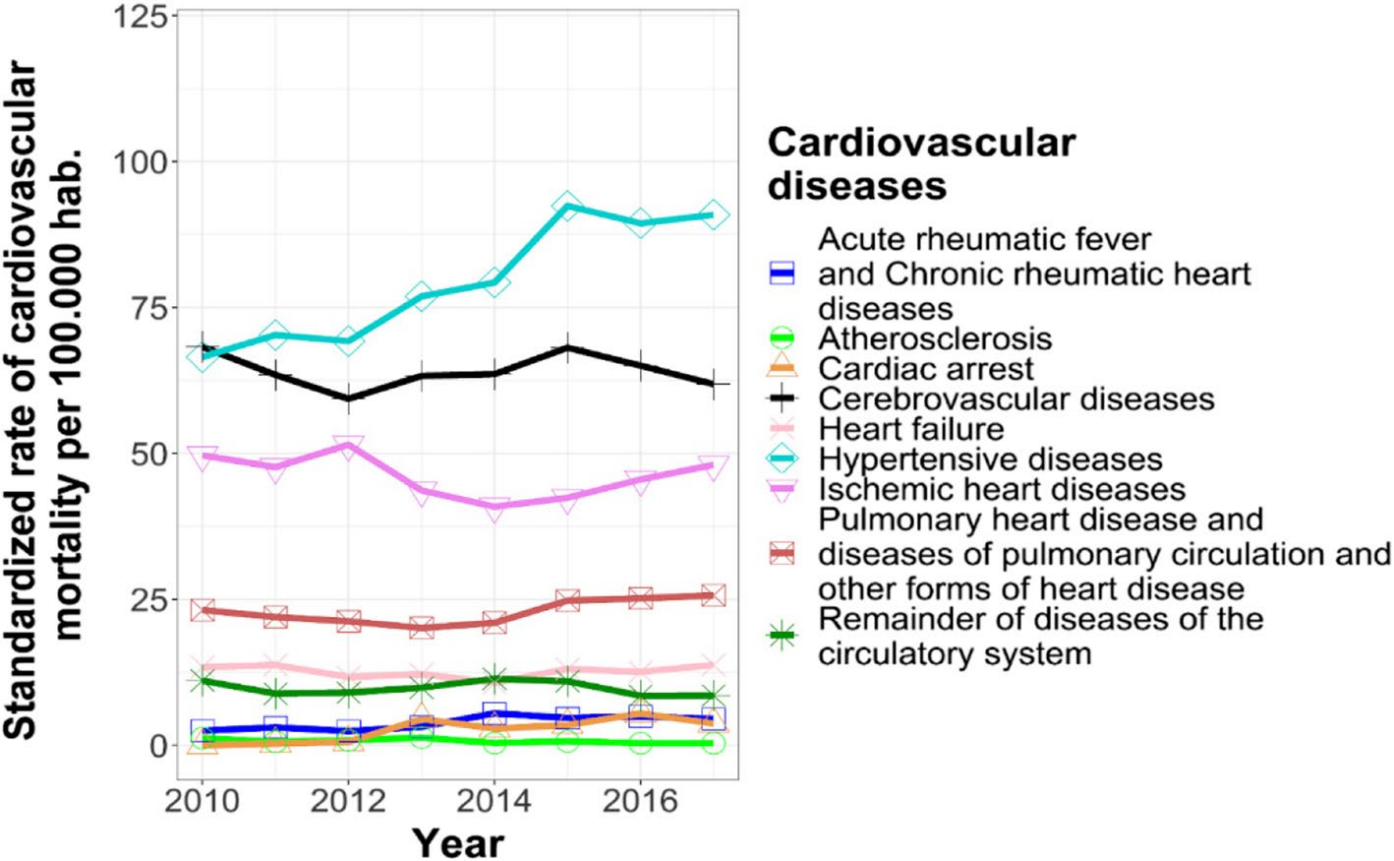
The age-standardized rates of cardiovascular mortality in Cali by type of cardiovascular disease from 2010 to 2017

From 2010 to 2017, there was an increase in the age-standarized mortality rate as the percentage change globally increased by 9.1% with a constant increase of 1,34% per year with a higher value in 2015. The age-standardized cardiovascular mortality rate of men was higher than that for women, peaking in 2016. For women, the age-standardized mortality rate of cardiovascular disease was lower than that for Cali reaching a peak in 2015. Between 2010 and 2017, the percent change in the direct standardized rate showed an increase of 10.38% and 9.30% for men and women respectively. On average, the age- standardized rate increased by 1.51% each year, for both men and women. See Table 1 and Fig. 2. The highest rate was due to hypertensive diseases followed by cerebrovascular diseases and ischemic heart diseases. See Fig. 3. Globally, this distribution was similar for both men and women. Of noticed, the age-standardized rates of cardiovascular mortality were higher in men compared to women over the period.

**Table 1:** Direct standardized rate of cardiovascular mortality in Cali, 2010–2017^a^

**Year**	**Standardized rate of cardiovascular mortality in Cali per 100.000 hab(IC 95%)**	**Standardized rate of cardiovascular mortality in men per 100.000 hab. (IC 95%)**	**Standardized rate of cardiovascular mortality in women per 100.000 hab. (IC 95%)**
2010	235.9 (227.7—244.5)	279.1 (265.0—293.7)	204.7 (194.7—215.1)
2011	230.0 (221.9—238.3)	262.6 (249.1—276.7)	206.2 (196.3—216.5)
2012	226.0 (218.1—234.2)	274.4 (260.7—288.7)	191.9 (182.5—201.7)
2013	235.1 (227.1—243.3)	277.9 (264.2—292.3)	205.3 (195.7–215.2)
2014	235.7 (227.8—243.8)	290.8 (276.7—305.4)	199.0 (189.8—208.6)
2015	260.7 (252.4—269.1)	309.8 (295.3 -325.0)	228.1 (218.4—238.2)
2016	257.1 (249.1—265.3)	319.5 (304.9—334.7)	216.4 (207.1—226.0)
2017	257.4 (249.5—265.5)	308.0 (249.1—322.5)	223.7 (214.3—233.4)

^a^The 2018 Cali population is the standard population

**Fig. 4:**
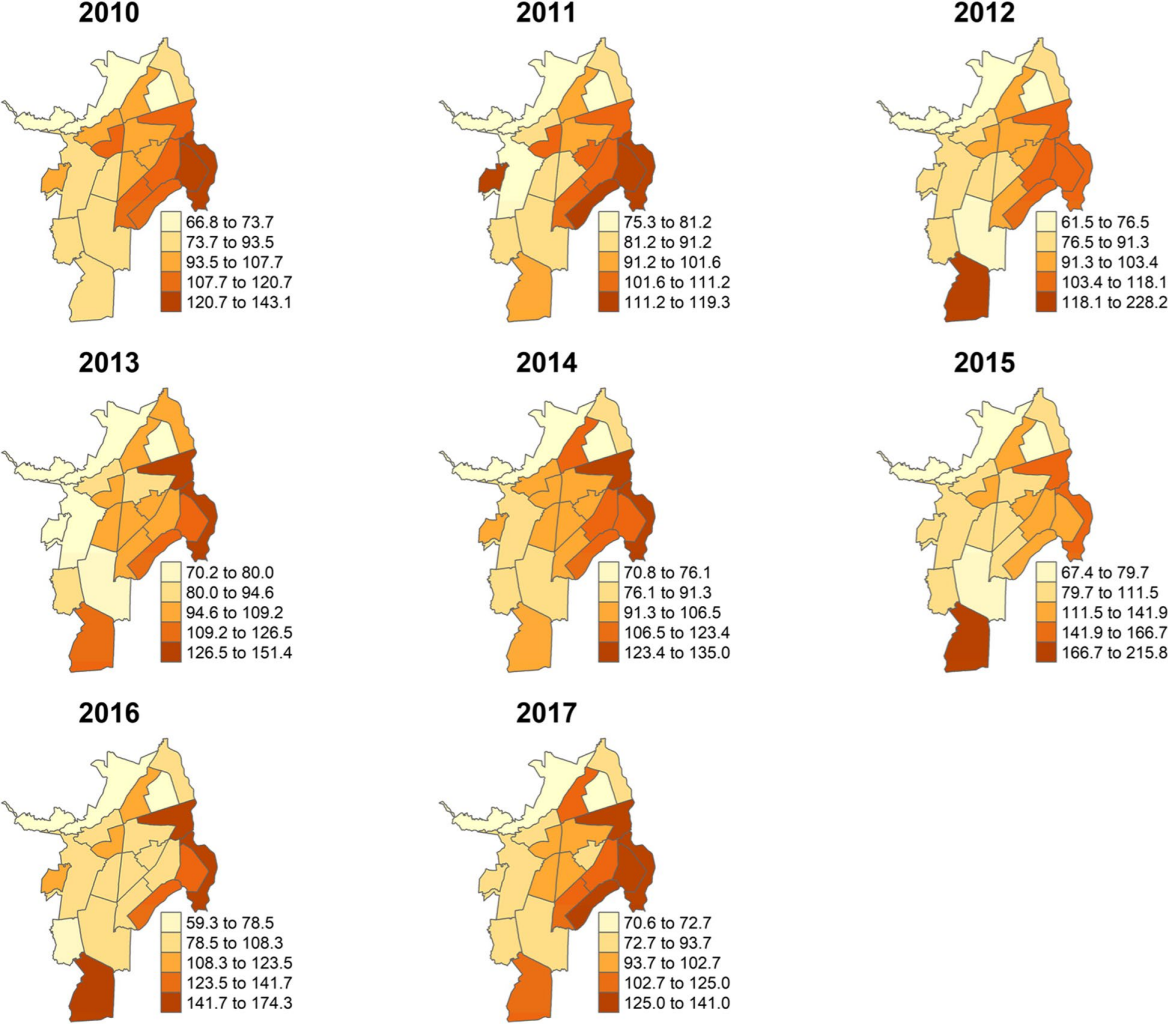
Smoothed standardized mortality ratio of cardiovascular mortality in Cali by district between 2010 and 2017

Globally, over the study period, the highest age-standardized rate of CVD mortality was observed in the district 22 located in the south of the city, followed by the district 21, 7, 14 and 15 located in the east and on the contrary, the districts 1 and 2 in the northwest of the city had the lowest SMR. Similarly, the results from measuring the smoothed SMR showed that the district 22 had the highest cardiovascular risk following by the districts 7, 13, 14, 15, 16 and 21. The lowest SMR were found in the districts 1 and 2. See Fig. 4

### Spatial analysis

**Fig. 5:**
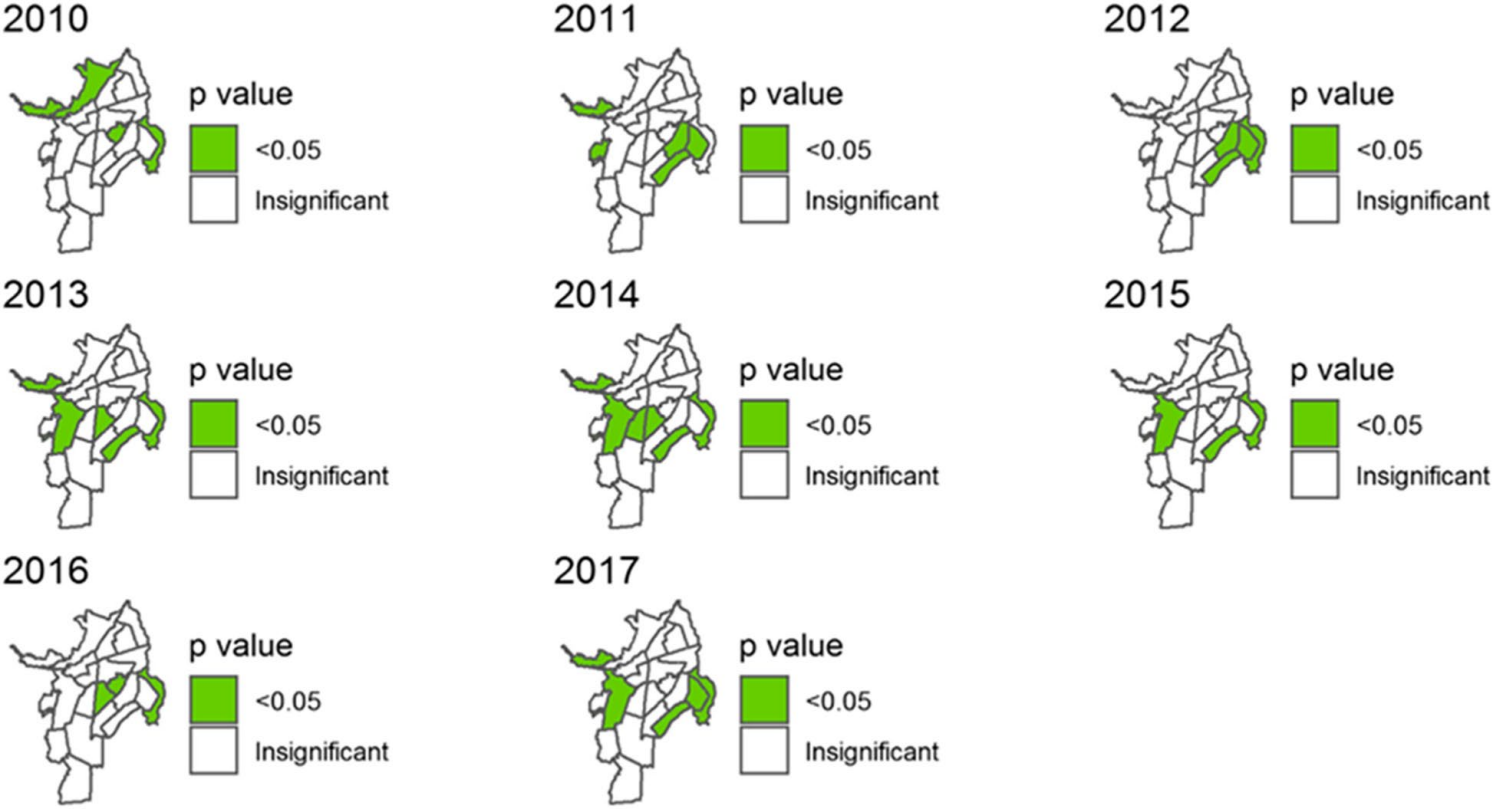
Significant spatial correlation according to Local Geary´s C Test for the standardized mortality ratio of cardiovascular mortality in Cali, from 2010 to 2017

The results of the Global Geary´s C test indicated that in each year, there was a significant spatial correlation (*p* < 0.05) in the standardized mortality ratio of cardiovascular diseases in Cali (see Table 2). During the period 2010–2017, the Local Geary´s C test identified clustering (*p* < 0.05), due mainly to the districts 1, 14, 15, 19 and 21 (See Table 3 and Fig. 5).

**Table 2:** Global spatial correlation in the standardized cardiovascular mortality ratio in Cali, from 2010 to 2017

**Year**								
**Geary’s**^a^	**2010**	**2011**	**2012**	**2013**	**2014**	**2015**	**2016**	**2017**
**C Test**	0.58235 (0.011)^a^	0.59917 (0.009)^a^	0.39417 (0.022)^a^	0.63923 (0.022)^a^	0.65112 (0.02)^a^	0.50728 (0.008)^a^	0.59687 (0.015)^a^	0.54108 (0.006)^a^

^a^Significant level *α* = 0.05

**Table 3:** Districts with local spatial correlation in the standardized cardiovascular mortality ratio in Cali, from 2010 to 2017, obtained by local Geary´s C test^a^

**Year**	**Districts**
2010	1,2,12,21
2011	1,13,14,15, 20
2012	13,14,15,21
2013	1,11,15,19,21
2014	1,10,11,15,19,21
2015	15,19,21
2016	11,12,21
2017	1,14, 15,19,21

^a^Significant level *α* = 0.05

Between 2010 and 2017, the district 1 had the lowest standardized ratio of cardiovascular mortality and the districts 14, 21 and 22 had the highest. Some characteristics of the deaths from the district 1 were analysed. Of notice, the district 21 had the highest percentage of premature mortality, 40%. Most deaths in the district 14 had subsidized health insurance, 67.7%, compared to 56.5% and 58.8% in the districts 1 and 21 respectively. See table S2 supplement.

## Discussion

Noncommunicable diseases were the main causes of mortality in Cali, Colombia, from 2010–2017, with cardiovascular diseases made up the highest proportion of deaths. Over the period, there was an increase of 9,11% in the age-standardized rate of cardiovascular mortality and nearly 30% of deaths were in those under 70 years of age. Noteworthy, the occurrence of cardiovascular deaths is spatially aggregated in some in districts at both low and higher of socio-economic status although the highest mortality cardiovascular ratio was in those at lower socio-economic status and located in peripheral districts.

Over the study period, there was an increase in the percentage change of age-standardized rate of cardiovascular mortality and and higher cardiovascular mortality rates were observed in men. This trend may be similar to the current trend in some high income countries [4] and similar to the Colombia variation in CVD and some low-middle income countries like India [6, 28, 29]. Variations in cardiovascular mortality are due to longer life expectancy, population growth and medical and preventive interventions [30]. The study period covers a period of seven years where the population remains stable with a roughly increase of 7% and the percentage of people over 60 years old slightly varied between 12% an 13% [18] so the constant increase may reflect the need to strength the preventive and treatment interventions. Worldwide evidence has shown that populations interventions aims at reducing smoking, unhealthy diet and physical inactivity as well as individual interventions with strengthen primary care services explained the decline in mortality from coronary heart disease [31]. For example, a reduction in stroke and ischemic mortality in Brazil attributed to the reforms in the primary care system [29].

The highest risk of of cardiovascular mortality was found in the districts 7, 13, 14, 15, 16, 21, and 22. All of them are located at the southeast of the city except for the districts 22 which at the southwest. Similar to observed worldwide population living in areas at lower socio-economic strata exhibit the highest risk of cardiovascular mortality which the case for districts 7,13, 14,15 16 and 21 locate at the southern of the city [12, 19]. However, population from district 22 is classified at the highest socio-economic status [19] so other mechanism are influencing the observed higher cardiovascular risk. As all the mentioned districts are peripherally located, one explanation could be related to the long time for receiving health care by those with a cardiovascular emergency. Complementary, as access to health care depends on the type of health insurance, it is possible that some institutions were not allowed to provided services to some individuals [32, 33]. This could be accounts for people living the district 22, which is close to the most complex health institution of the city. Clearly, the closest the better, as the long time between acute symptoms and receiving health care increases cardiovascular mortality [34, 35]. Of notice, nearly 60% of people living in the southeast are covered by subsidize insurance supported by public funding and almost 100% of of residents in the district 22 belong to contributive insurance supported by private resources from formal workers [36, 37]. Although some evidence has shown that people covering by subsidized insurance has more barriers to access health services [38], this results show light on unidentified limitations to receive health care for those with acute cardiovascular conditions. [32].

Over the period, clustering was mainly identified in the districts 1,2, 14,15,19 and 21. The districts 1 and 2 had the lowest and the districts 14, 15, 19, and 21 the highest SMR. The districts 1, 14, 15, 19 and 21 are the lowest socio-economic strata comparing to the district 2 which is at the highest strata [37]. As mentioned by other authors, the conditions related to the neighbourhood surpass the concept of socio-economic strata [11, 39]. Then, these results could unveil the contribution of other factors which has not been identified. The green space has also associated with physical activity however in Cali, Hong A et al. found that this relation in those at higher socio-economic strata [40]. Pollution has clearly linked to cardiovascular mortality rate [13], some local environmental reports has shown excess of polluted particles in the north of the city [41] and the districts in south-eastern are nearby to the former local landfill which was closed in 2008 due to environment impact [42]. Finally, as the prevalence of cardiovascular risk factors was not measured by district, we could not estimate its local impact.

Hypertensive diseases following by cerebrovascular diseases and ischaemic heart diseases were main causes of cardiovascular mortality. By contrast, ischaemic heart disease accounted for the most of cardiovascular mortality in Colombia over the same period according to the Ministry of Health and Social Protection report [28]. However, analysis of Colombian data has been shown that the prevalence of hypertension and hypertension related disease have been increased since 1990 nearly by 30% and 105% respectively [6, 43]. Complementary, the 2015 Colombian SABE survey found that 63% of people aged 60 years and over suffer from hypertension in Cali [44]. Therefore, the results suggest that the prevention and control of hypertension potentially has the biggest impact in reducing the cardiovascular mortality in Cali. Of notice, we did not measure mortality for external causes which has been associated with high impact particular in younger male [45].

As expected, men had higher rates of cardiovascular mortality than women. Worldwide data has been shown that the risk of coronary heart mortality is higher in men than women particularly at young and middle age. However, in older people the men-women risk ratio of cardiovascular mortality becomes less with stroke accounting for most of the mortality in women [13]. Estrogen, variations in biomarkers and inflammatory mechanism between women and men account for this difference although the explanations for this difference have not been totally sorted out [46].

### Study strength and limitations

The study measured the spatial distribution of CVD mortality by using smoothing SMR which is needed for small areas as it is pointed out by Waller & Gotway [25]. Complementary, the spatial correlation was analysed by means of three different indexes. The Moran *I* test and the Bayesian empirical (*BEI*) test were sensitive to extreme standardized mortality ratio values so that the results provided by them did not allow to evaluate the true significance for spatial autocorrelation in all years of study. By contrast, the Geary *C* test, calculated as the differences between the variables in the different zones, was not affected by extreme values and then it was considered the most appropriate measure for the spatial autocorrelation of mortality risks of cardiovascular disease in Cali.

The National register of statistics has consolidated the methods to collect the mortality cases which have been assessed by local and international researcher as one provided with high quality data [47, 48]. In the current analysis, only 2,54% of the deaths did not have information on place of living so this data was not recorded and the cause of death was register by physician for all registers. In addition, there was no data about the distribution of population for the 15 townships located in rural areas so that epidemiological and spatial analyses did no carry out for this area. Finally, there was not spatial aggregation in the district 22 may be due the low density of population in this area [37].

In conclusion, the study agrees with the worldwide mortality reported by the Institute for the Institute for Health Metrics and Evaluation in 2019 and the Colombian reports that cardiovascular mortality is the main cause of mortality. The current analysis added two important points for Cali, the main cause of cardiovascular mortality is due to hypertensive diseases and the risk of cardiovascular mortality is heterogenous with highest mortality rates in districts located in peripheral areas and moreover the deaths are spatially aggregated regardless of socio-economic status. These findings suggest that those population living in peripheral may have more limitations in access to acute health care and there could be some barriers to receive promptly health care for emergency situations.

## Supplementary Information

**Additional file 1: Table S1.** Characteristics of deaths due to cardiovascular diseases registered in Cali between 2010 and 2017. **Table S2.** Characteristics of death from districts 1, 14 and 21 in Cali, between 2010 and 2017.

## Data Availability

The mortality register is national data collection established by the Department of National Statistics. There is a national format which must be filled by physicians or health authority where there is no physician to bury a person. The first compilation of the formats is done by the Secretary of Health at each municipality in the country. The data is available to the links http://microdatos.dane.gov.co/index.php/catalog/MICRODATOS/about_collection/22/5 and http://rssvr2.sispro.gov.co/reportesAsis/.
