# Supplementary material for: Spatial variation of cardiovascular mortality in Cali, Colombia, between 2010 and 2017
Source: BMC Public Health. 2023 Mar 31;23:616. doi: 10.1186/s12889-022-14907-x (PMC10064751; doi:10.1186/s12889-022-14907-x)
Supplement: Supplementary file 1 — Additional file 1: Table S1. Characteristics of deaths due to cardiovascular diseases registered in Cali between 2010 and 2017. Table S2. Characteristics of death from districts 1, 14 and 21 in Cali, between 2010 and 2017. [file 12889_2022_14907_MOESM1_ESM.docx]

| **Spatial distribution of cardiovascular mortality in Cali, Colombia, analysis of regional register between 2010 and 2017**  Table S1.  Characteristics of deaths due to cardiovascular diseases registered in Cali between 2010 and 2017 | | | | | | | | | | | | | | | | | | | | | | | | |  |
| --- | --- | --- | --- | --- | --- | --- | --- | --- | --- | --- | --- | --- | --- | --- | --- | --- | --- | --- | --- | --- | --- | --- | --- | --- | --- |
|  |  |  |  |  |  |  |  |  |  |  |  |  |  |  |  |  |  |  |  |  |  |  |  |  |  |
|  |  |  |  |  |  |  |  |  |  |  |  |  |  |  |  |  |  |  |  |  |  |  |  |  |  |
|  |  |  |  |  |  |  |  |  |  |  |  |  |  |  |  |  |  |  |  |  |  |  |  |  |  |
|  |  |  |  |  |  |  |  |  |  |  |  |  |  |  |  |  |  |  |  |  |  |  |  |  |  |
| **Characteristics** | **2010** | |  | **2011** |  |  | **2012** | |  | **2013** | |  | **2014** | |  | **2015** | |  | **2016** |  |  | **2017** | | **Total** |  |
|  |  |  |  |  |  |  |  |  |  |  |  |  |  |  |  |  |  |  |  |  |  |  |  |  |  |
| **Sex** |  |  |  |  |  |  |  |  |  |  |  |  |  |  |  |  |  |  |  |  |  |  |  |  |  |
| Men | 1579 | 49,3 |  | 1526 | 47,6 |  | 1609 | 50,1 |  | 1653 | 48,3 |  | 1746 | 49,3 |  | 1872 | 47,1 |  | 1987 | 49,0 |  | 1997 | 47,7 | 13969 | 48,5 |
| Women | 1620 | 50,6 |  | 1680 | 52,4 |  | 1605 | 49,9 |  | 1769 | 51,7 |  | 1793 | 50,7 |  | 2106 | 52,9 |  | 2069 | 51,0 |  | 2192 | 52,3 | 14834 | 51,5 |
| No data | 1 |  |  | 0 |  |  | 0 |  |  | 0 |  |  | 0 |  |  | 0 |  |  | 0 |  |  | 0 |  | 1 |  |
|  | 3200 |  |  | 3206 |  |  | 3214 |  |  | 3422 |  |  | 3539 |  |  | 3978 |  |  | 4056 |  |  | 4189 |  | 28804 |  |
|  |  |  |  |  |  |  |  |  |  |  |  |  |  |  |  |  |  |  |  |  |  |  |  |  |  |
| **Age** | **Number** | **%** |  | **Number** | **%** |  | **Number** | **%** |  | **Number** | **%** |  | **Number** | **%** |  | **Number** | **%** |  | **Number** | **%** |  | **Number** | **%** | **Number** | **%** |
| Mean ± SD | 74.3 | ± 0.51 |  | 74.6 | ± 0.53 |  | 75.8 | ± 0.49 |  | 75.8 | ± 0.49 |  | 75.9 | ± 0.47 |  | 76.5 | ± 0.46 |  | 76.6 | ± 0.44 |  | 76.0 | ± 0.45 | 75.8 | ± 0.17 |
| Premature death (30 to 69 years) | 1008 | 31.5 |  | 917 | 28.6 |  | 880 | 27.4 |  | 935 | 27.3 |  | 978 | 27.6 |  | 1022 | 25.7 |  | 1060 | 26.1 |  | 1115 | 26.6 | 7915 | 27,5 |
| Premature death in men (30 to 69 years) | 604 | 38.3 |  | 528 | 34.6 |  | 554 | 34.4 |  | 545 | 32.9 |  | 593 | 33.9 |  | 591 | 31.6 |  | 638 | 32.1 |  | 655 | 32.8 | 4708 | 16,3 |
| Premature death in women (30 to 69 years) | 404 | 29.4 |  | 389 | 23.2 |  | 326 | 20.3 |  | 390 | 22.1 |  | 385 | 21.4 |  | 431 | 20.5 |  | 422 | 20.4 |  | 460 | 20.9 | 3207 | 11,1 |
|  |  |  |  |  |  |  |  |  |  |  |  |  |  |  |  |  |  |  |  |  |  |  |  |  |  |
| **Marital status** | **Number** | **%** |  | **Number** | **%** |  | **Number** | **%** |  | **Number** | **%** |  | **Number** | **%** |  | **Number** | **%** |  | **Number** | **%** |  | **Number** | **%** | **Number** | **%** |
| Widowed | 1011 | 31,6 |  | 1029 | 32,1 |  | 1042 | 32,4 |  | 1163 | 34,0 |  | 1193 | 33,7 |  | 1410 | 35,4 |  | 1313 | 32,4 |  | 1406 | 33,6 | 9567 | 33,2 |
| Married | 795 | 24,8 |  | 819 | 25,5 |  | 816 | 25,4 |  | 830 | 24,3 |  | 817 | 23,1 |  | 896 | 22,5 |  | 986 | 24,3 |  | 934 | 22,3 | 6893 | 23,9 |
| Never married | 838 | 26,2 |  | 825 | 25,7 |  | 850 | 26,4 |  | 884 | 25,8 |  | 930 | 26,3 |  | 1108 | 27,9 |  | 1203 | 29,7 |  | 1275 | 30,4 | 7913 | 27,5 |
| Divorced | 192 | 6,0 |  | 181 | 5,6 |  | 178 | 5,5 |  | 152 | 4,4 |  | 215 | 6,1 |  | 211 | 5,3 |  | 221 | 5,4 |  | 228 | 5,4 | 1578 | 5,5 |
| No data | 364 | 11,4 |  | 352 | 11,0 |  | 328 | 10,2 |  | 393 | 11,5 |  | 384 | 10,9 |  | 353 | 8,9 |  | 333 | 8,2 |  | 346 | 8,3 | 2853 | 9,9 |
|  | 3200 |  |  | 3206 |  |  | 3214 |  |  | 3422 |  |  | 3539 |  |  | 3978 |  |  | 4056 |  |  | 4189 |  |  |  |
|  |  |  |  |  |  |  |  |  |  |  |  |  |  |  |  |  |  |  |  |  |  |  |  |  |  |
| **Education** | **Number** | **%** |  | **Number** | **%** |  | **Number** | **%** |  | **Number** | **%** |  | **Number** | **%** |  | **Number** | **%** |  | **Number** | **%** |  | **Number** | **%** |  |  |
| Basic | 2005 | 62,7 |  | 2062 | 64,3 |  | 2053 | 63,9 |  | 2166 | 63,3 |  | 2237 | 63,2 |  | 2558 | 64,3 |  | 2611 | 64,4 |  | 2622 | 62,6 | 18314 | 63,6 |
| Secondary | 400 | 12,5 |  | 379 | 11,8 |  | 390 | 12,1 |  | 387 | 11,3 |  | 416 | 11,8 |  | 513 | 12,9 |  | 498 | 12,3 |  | 496 | 11,8 | 3479 | 12,1 |
| Technic, professional and others | 167 | 5,2 |  | 135 | 4,2 |  | 170 | 5,3 |  | 173 | 5,1 |  | 174 | 4,9 |  | 217 | 5,5 |  | 215 | 5,3 |  | 495 | 11,8 | 1746 | 6,1 |
| No data | 628 | 19,6 |  | 630 | 19,7 |  | 601 | 18,7 |  | 696 | 20,3 |  | 712 | 20,1 |  | 690 | 17,3 |  | 732 | 18,0 |  | 576 | 13,8 | 5265 | 18,3 |
|  | 3200 | 100,0 |  | 3206 | 100,0 |  | 3214 | 100,0 |  | 3422 | 100,0 |  | 3539 | 100,0 |  | 3978 | 100,0 |  | 4056 | 100,0 |  | 4189 | 100 | 28804 | 100,0 |
|  |  | 0 |  |  |  |  |  |  |  |  |  |  |  |  |  |  |  |  |  |  |  |  |  |  |  |
| **Health insurance** |  |  |  |  |  |  |  |  |  |  |  |  |  |  |  |  |  |  |  |  |  |  |  |  |  |
| Contributive | 1544 | 48,3 |  | 1608 | 50,2 |  | 1661 | 51,7 |  | 1705 | 49,8 |  | 1739 | 49,1 |  | 2147 | 54,0 |  | 2178 | 53,7 |  | 2117 | 50,5 | 14699 | 51,0 |
| Subsidized | 1077 | 33,7 |  | 1087 | 33,9 |  | 1136 | 35,3 |  | 1256 | 36,7 |  | 1360 | 38,4 |  | 1452 | 36,5 |  | 1518 | 37,4 |  | 1700 | 40,6 | 10586 | 36,8 |
| Uninsured | 455 | 14,2 |  | 420 | 13,1 |  | 328 | 10,2 |  | 362 | 10,6 |  | 294 | 8,3 |  | 270 | 6,8 |  | 217 | 5,4 |  | 179 | 4,3 | 2525 | 8,8 |
| Others | 124 | 3,9 |  | 91 | 2,8 |  | 89 | 2,8 |  | 98 | 2,9 |  | 144 | 4,1 |  | 106 | 2,7 |  | 143 | 3,5 |  | 187 | 4,5 | 982 | 3,4 |
| No data |  |  |  |  |  |  |  |  |  | 1 | 0,0 |  | 2 | 0,1 |  | 3 | 0,1 |  |  | 0,0 |  | 6 | 0,1 | 12 | 0,0 |
|  | 3200 | 100,0 |  | 3206 | 100,0 |  | 3214 | 100,0 |  | 3422 | 100 |  | 3539 | 100 |  | 3978 | 100 |  | 4056 | 100 |  | 4189 |  | 28804 | 100,0 |

**Table S2 Characteristics of death from districts 1, 14 and 21 in Cali, between 2010 and 2017**

| **Characteristics** | **District 1** | **District 14** | **District 21** |
| --- | --- | --- | --- |
|  | **Percentage** | **Percentage** | **Percentage** |
| **Sex** |  |  |  |
| Men | 42.3 | 49.4 | 42.9 |
| Women | 57.7 | 50.6 | 57.1 |
|  |  |  |  |
|  |  |  |  |
| **Premature death (30 to 69 years)** | 25.9 | 27.0 | 40.0 |
|  |  |  |  |
| **Marital status** |  |  |  |
| Widowed | 43.5 | 33.4 | 29.4 |
| Married | 18.2 |  |  |
| Never married |  | 24.9 | 22.7 |
|  |  |  |  |
|  |  |  |  |
|  |  |  |  |
|  |  |  |  |
| **Education** |  |  |  |
| Basic | 52.9 | 49.4 | 52.1 |
| **Health insurance** |  |  |  |
| Contributive | 36.1 | 25.7 | 36.1 |
| Subsidized | 56.5 | 67.7 | 58.8 |
| No data | 7.4 | 6.6 | 5,1 |
|  |  |  |  |
